# Effect of masks on speech intelligibility in auralized classroomsa)

**DOI:** 10.1121/10.0002450

**Published:** 2020-11-24

**Authors:** Pasquale Bottalico, Silvia Murgia, Giuseppina Emma Puglisi, Arianna Astolfi, Karen Iler Kirk

**Affiliations:** 1 Department of Speech and Hearing Science, University of Illinois, Urbana-Champaign, Champaign, Illinois 61820, USA; 2 Dipartimento Energia, Politecnico di Torino, Torino, Italy

## Abstract

This study explored the effects of wearing face masks on classroom communication. The effects of three different types of face masks (fabric, surgical, and N95 masks) on speech intelligibility (SI) presented to college students in auralized classrooms were evaluated. To simulate realistic classroom conditions, speech stimuli were presented in the presence of speech-shaped noise with a signal-to-noise ratio of +3 dB under two different reverberation times (0.4 s and 3.1 s). The use of fabric masks yielded a significantly greater reduction in SI compared to the other masks. Therefore, surgical masks or N95 masks are recommended in teaching environments.

## I. INTRODUCTION

Due to the recent pandemic event related to Covid-19, many countries are mandating the use of face masks to reduce the spread of the virus. Unfortunately, those masks can have a detrimental effect on speech communication at both ends as listeners will probably experience a decrease in intelligibility and speakers will experience an increase in vocal effort.

Face masks consist of multilayered fabrics, made of natural fibers such as cotton or synthetic materials such as polypropylene, polyester, or polyurethane. Fabrics are often used for sound absorption due to their porous structure. A porous material absorbs sound energy as it dampens the oscillation of the air particles through friction. Porous absorbers are most effective in the high-frequency range. As a consequence, face masks act as a low-pass filter that attenuates speech intensity, mainly at mid-to-high frequencies that are fundamental for speech comprehension. For example, the most relevant frequency range for speech intelligibility (SI) is 0.5–4 kHz,^1^ whereas the sound attenuation of face masks is greatest in the frequency range between 2 and 8 kHz.^2^ The negative effects of wearing a face mask on SI could be even worse in poor acoustic conditions, such as in the presence of high reverberation time and background noise. Those environmental factors already are a challenge for speech communication in schools.

A secondary effect of wearing face masks is the loss of lip reading and visual speech cues from facial expressions. This secondary effect is particularly detrimental to people with hearing loss. Elimination of visual speech reading cues can decrease SI by as much as 20% for people with moderate bilateral sensorineural hearing loss.^3^

Mendel *et al.*^4^ studied the effect on SI of masks used in relation to the healthcare environment. In their experiment, 1 speaker, 15 normally hearing listeners, and 15 listeners with hearing loss participated. The hearing loss group had varying degrees and configurations of hearing loss with thresholds equal to or poorer than 25 dB hearing level (HL) for the octave frequencies from 250 to 8000 Hz. The test consisted of eight ten-sentence lists from the Connected Speech Test (CST).^5^ Two sets of stimuli were recorded by an adult male talker, with and without a surgical mask. The two sets of stimuli were presented under two conditions: in quiet and in the presence of dental office noise (noise from a dental hand drill) at a signal-to-noise ratio (SNR) of +5 dB. The results did not show an effect of the masks on SI, likely due to the listening conditions which should present variable noise levels. The scores showed a ceiling effect with intelligibility higher than 96.9% in all of the conditions.

Different results were presented by Wittum *et al.*^6^ The test performed by the authors consisted of eight lists of phrases from the Speech Perception in Noise Test (SPIN).^7^ The lists were recorded by a male and a female professional speaker under three different conditions: without wearing a mask, while wearing a surgical mask, and while wearing a face shield together with the surgical mask. The speech material was delivered with a multi-talker babble noise at an SNR of +4 dB. The results from six listeners showed that with the same SNR, the percentage of correct responses was highest for the unmasked condition, followed by the masked condition, and the mask plus shield condition. However, from their experiment, it appeared that the signal-to-masker ratio was set a bit too high, leading to a performance in the unmasked condition that neared the ceiling at 100%. For this reason, the authors suggested using a lower SNR in future studies to avoid such a ceiling effect.

Saeidi *et al.*^8^ presented a preliminary study examining the effect of different face masks on speech acoustics. They compared speech recordings (readings and spontaneous speech) from four male and four female speakers without face covers to speech recordings of the same speakers with four different face covers: a motorcycle helmet, a rubber mask, a surgical mask, and a hood with a scarf. For each of the conditions, each speaker was asked to read a set of sentences and choose an image to describe in order to simulate spontaneous speech. Their analysis of the medium-to-long term spectrum of speech under each condition identified the surgical mask and the combination of hood and scarf as more degrading conditions for speech above 1 kHz than the other two types of face covers.

The effect of masks on an objective SI parameter, the Speech Transmission Index (STI), was evaluated by Palmiero *et al.*^9^ Three masks were included: a surgical mask, an N95 mask, and an elastomeric half-mask air-purifying respirator (EAPR). STI was measured under two different experimental conditions: (1) STI measurements using a modified version (3 samples × 5 replicate measurements) of the National Fire Protection Association (NFPA) 1981 standard and SNR of −15 (66 dBA) and (2) STI measurements utilizing modified pink noise levels of 52.5 dBA (−2 SNR) to 72.5 dBA (+7 SNR) in 5.0 dBA increments. The results showed that under the same SNR conditions, the EAPR appears to have the most degrading effect on SI (poor/fair STI range), followed by the N95 mask (good STI range), and then the surgical mask (excellent STI range). The surgical mask condition yielded the highest STI value.

The study results summarized above highlight the need to investigate more thoroughly the use of masks on SI, especially in school environments where understanding is fundamental to learning and academic success. Furthermore, the effects of masks on SI should be studied in relation to other factors influencing speech transmission, such as the communication path between speakers and listeners, e.g., the SNR and reverberation time.^9–11^

Reverberation degrades speech prosody as temporal smearing inhibits the correct identification of cues, such as duration and rhythm, that convey prosodic information.^12^ High noise levels can degrade the speech signal by decreasing the perceived sound level, thereby reducing SI.^9–11^

Due to the spread of the Covid-19 virus, it is likely that teachers and professors in the majority of schools all over the world will wear face masks. This challenge for speech communication will be added to already existing negative factors, such as poor acoustics and high noise levels, often experienced in classrooms. Therefore, this study is intended to contribute first insights on the topic by addressing the following research questions:

(1) Which type of face mask yields the highest level of SI in simulated classroom conditions with low and high reverberation times?
(2) Which type of face mask yields the lowest level of listening effort (LE) in simulated classroom conditions with low and high reverberation times?

The choice of which type of mask to use depends on other factors as well; however, the goal of this study is to understand the potential impact on SI of various masks. This could aid teachers in making informed decisions regarding mask use.

## II. EXPERIMENTAL METHOD

### A. Participants

Forty college students from the University of Illinois at Urbana-Champaign were enrolled in the study. From the results of Mendel *et al.*,^4^ the effect size of the difference in intelligibility when the speaker was wearing a surgical mask compared to a no mask condition was 0.76. We estimated a sample size of 40 listeners to obtain a statistical power of 80% with a significance level of *p* < 0.001, using four different mask conditions. The inclusion criteria were (1) currently enrolled as a student at the University of Illinois; (2) the ability to use headphones during the experiment; (3) no reported history of speech, language, and/or hearing problems; and (4) a native English speaker or advanced proficiency in English by self-report.

Before the listening test, some information was collected from the listeners and is summarized here. The participants were equally divided between undergraduate and graduate students. Twenty percent of the participants were male and 80% were female. All of them reported performing the test at home; 90% rated the background noise in their home as quiet or very quiet. Among the participants, 90% were native English speakers, whereas 10% reported advanced proficiency in English. The listeners were asked to report the model of the headphones used during the intelligibility test. Among the 40 listeners, 22 used Apple headphones (Cupertino, CA), five used Sony headphones (Tokyo, Japan), and three were unsure as to the model. The remaining headphone types (each used by only one participant) were JVC (Yokohama, Japan), Bose (Farmingham, MA), Symphonized (Brooklyn, NY), Beats (Cupertino, CA), Vogek (Houston, TX), Jabra Letsfit (Copenhagen, Denmark), Nool (Brooklyn, NY), Samsung (Seoul, South Korea), and Cowin (City of Industry, CA). The type of headphone was requested because different headphones have different frequency responses, introducing a confounding variable. All participants were aware of the purpose of the test and provided with a document containing instructions on how to run the test. All participants signed an online informed consent for their participation in the experiment, which was approved by the Institutional Review Board of the University of Illinois Urbana-Champaign under Protocol No. 20209.

### B. Speech stimuli

The speech stimuli consisted of the first eight lists of the consonant-nucleus-consonant (CNC) word test. The CNC word test consists of lists of monosyllabic words with equal phonemic distribution across lists. Each list exhibits approximately the same phonemic distribution as the English language.^13^ The original CNC lists were revised to eliminate relatively rare words and proper nouns.^14^ Each of the 10 lists constituted a set of 50 words containing approximately the same set of phonemes. The phonemes are distributed proportionally to the phonemic structure of English words occurring with a minimum frequency of one per million according to the Thorndike and Lorge frequency count.^15^

The recordings for the CNC word test are commercially available^16^ and consist of 500 test words organized into 50-word lists. Each word is preceded by the carrier word “ready.” The speech material is recorded by a male speaker with a standard American English dialect.

**FIG. 1.**
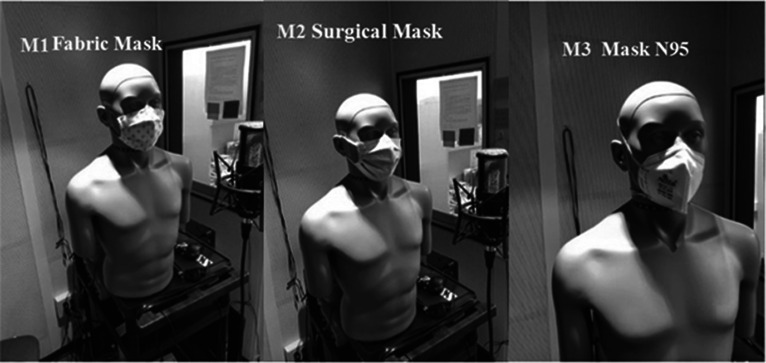
Head and torso simulator (HATS) wearing the three masks used in the study.

The recordings from the compact disc (CD)^16^ were played in a sound booth by a Head and Torso Simulator with Mouth Simulator (HATS, 45BC KEMAR, GRAS, Holte, Denmark) four times: (*M*0) without wearing a mask, (*M*1) while wearing a fabric mask, (*M*2) while wearing an N95 mask, and (*M*3) while wearing a surgical mask. The fabric mask consisted of two layers of cotton with a third layer of activated carbon between them; the other two masks were standardized products. Figure 1 shows the HATS wearing the three types of masks used for the recording. The recordings of the speech emitted by the HATS were performed with a microphone P120 (AKG, Vienna, Austria) placed in front of the mouth at 30 cm and connected to a personal computer (PC) through a soundboard UH7000 (TASCAM, Montebello, CA).

Two different room acoustic conditions were considered in terms of reverberation time. Binaural room impulse responses (BRIRs) were measured in two classrooms that presented different architectural features, dimensions, volumes, and, thus, acoustics. Room 1 had a volume of 171 m^3^ and presented optimal acoustic conditions with a reverberation time at mid-frequencies (0.5–1 kHz) of 0.4 s. The room was subject to an acoustic treatment that included a false ceiling made of rock-wool panels, a reflective panel placed above the teacher's desk to re-direct the useful reflections to the rear side of the room, and a mix of absorbent and vibrating panels on the lateral walls. Room 2, with a volume of 282 m^3^, was not subject to any acoustic treatment and exhibited a reverberation time at mid-frequencies of 3.1 s. Measurements were performed at the end of the school year when desks and chairs were removed for general cleaning of the rooms. Therefore, only shelves on the walls were present at that time, and it should be considered that all of the following results are related to a fully unoccupied classroom condition. The sound source that was used to emit an exponential sweep signal (3 s duration, 0.5–2 kHz range, three repetitions with 5 s silent intervals in between) was a TalkBox (by NTi Audio, Schaan, Liechtenstein) that has the same polar directivity diagram of the human voice. The receiver was a HATS (model 4128 by Brüel and Kjær, Nærum, Denmark). The source-to-receiver distance used in the experiment represented the typical distance between an ideal teacher and a student seated in the first row of seats, i.e., 1.5 m. This distance was applied in order to make the two classrooms comparable in terms of distance from the source but also to consider that reverberation affects the listening perception in room 2 given the much higher reverberation time. As such distance, 1.5 m, is approaching the critical distance (i.e., the distance from the sound source where the direct and reverberant sound energies become equal)^17^ of 1.2 m in room 1, and it is much higher than the critical distance of 0.5 m in Room 2, it is expected that at the receiver position the reverberated sound affects the perception more in room 2 than in room 1. In this way, the differences in SI can be limited to reverberation and the external factors used as input in the study (i.e., use and type of face mask).

The acquired sweep signals were convolved with the inverse filter of the originally emitted sweeps to obtain the BRIR that was used in the current study. Such BRIRs were convolved with the recorded speech stimuli to simulate the different environments using matlab version R2017a (The MathWorks, Natick, MA). The mono-channel speech material was convolved separately with the two channels of the two oral binaural impulse responses, and the resulting files were combined in a stereophonic audio file.

From the ten CNC word lists, only eight were used. Lists 1 and 5 were used for the recordings in which the speaker did not wear a mask; lists 2 and 6 were used for the recordings with the fabric mask (*M*1), lists 3 and 7 were used for the recordings with the surgical mask (*M*2) and lists 4 and 8 were used for the recordings with the N95 mask (*M*3). All of the previously listed conditions were then convolved with the two BRIRs. A whole list was assigned to a specific condition because each list contains the same phoneme distribution; however, each CNC stimulus was presented in a random order independently from the list. After the convolution, speech-shaped-noise was added to the speech material with a SNR of +3 dB. The 50 words in each list were then segmented 1 by 1 and presented to the listeners in random order.

### C. Procedure

The test was administered online using the platform SurveyGizmo because of the interruption of face-to-face research imposed by the pandemic emergency. Each participant was expected to listen to a total of 400 stimuli consisting of a combination of 8 lists of 50 words. The eight lists included four mask conditions per two-room acoustic conditions, all of them mixed with a speech-shaped noise at +3 dB of SNR. This level of SNR was chosen based on data reported in the literature.^4–6^ Pilot data on ten listeners were collected to measure the performance across the conditions. This was done to ensure that the SNR did not produce ceiling or floor effects.

Each subject was able to access the test individually and conducted it from his/her computer using headphones. They conducted speech recognition testing in an environment that they reported as containing a low level of background noise. Because the test was administered online, each participant was able to decide on the appropriate volume.

After reading the consent form and instructions, each participant could run the test. When they pressed the “start” button, the first stimulus was played. The CNC stimuli were presented in random order independently from the reverberation or the mask conditions. The listener then had to type the word played. Prior to continuing with the next stimulus item, the participant had to assess the degree of LE required to perceive the word. This was measured using a visual analog scale from 0 to 100 corresponding to “extremely difficult” and “extremely easy.” Next, the survey platform recorded the typed word and the LE rating for each stimulus. The whole test lasted about one hour, and the participants received a gift certificate of $15 in payment for their participation.

### D. Sound attenuation of the masks

**FIG. 2.**
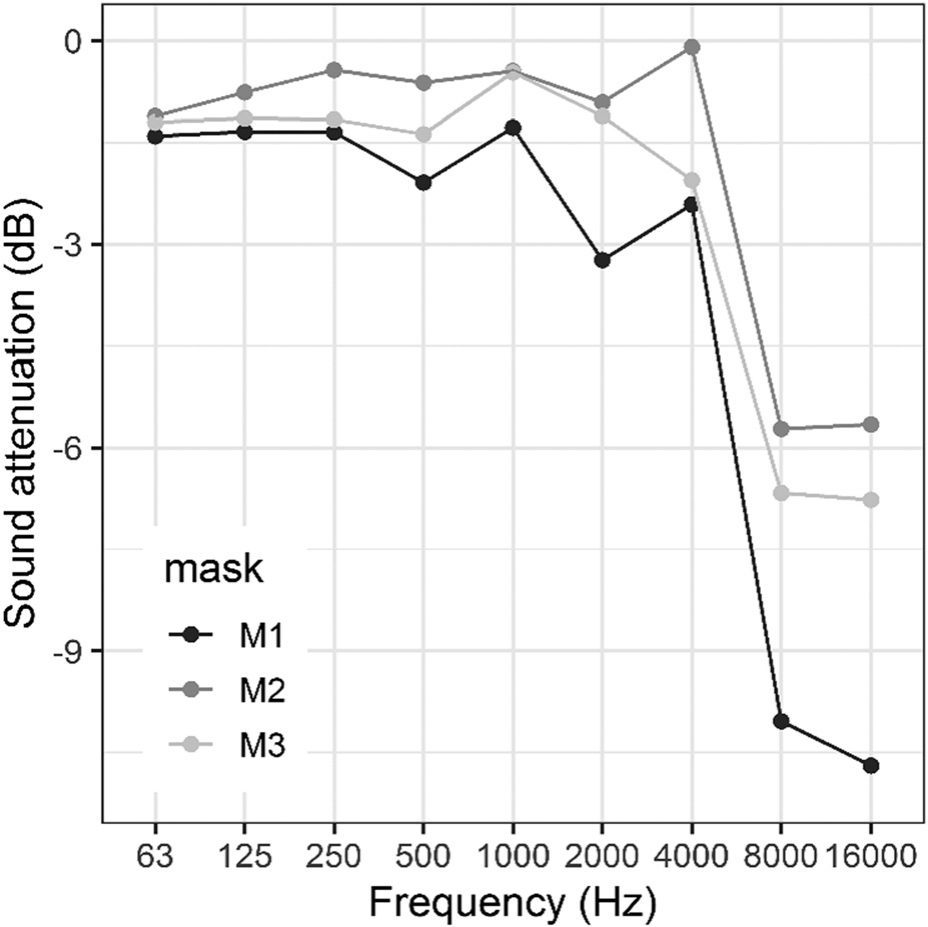
Sound attenuation introduced by the different types of masks per octave band. (M1 = fabric mask, M2 = surgical mask, M3 = N95 mask).

The masks served as a low-pass filter for the speech stimuli presented to the participants. The attenuation associated with each mask type was obtained by measuring the sound pressure levels per octave band of pink noise emitted by the HATS without a mask and subtracting them from the sound pressure levels recorded while the HATS was emitting the same pink noise and wearing the three masks used in the experiment. The pink noise levels were measured with a measurements microphone M2211 (Class 1 frequency response, NTi Audio Inc., Tigard, OR) placed at 0.5 m in front of the HATS mouth and analyzed by means of an NTI XL2 Audio and Acoustic Analyzer. The attenuation per octave band of each mask is shown in Fig. 2. High frequencies (from 2 to 16 kHz) are the most attenuated for all the masks. The fabric mask (*M*1) produced the greatest attenuation with an overall attenuation of 4.2 dB across the octave bands from 63 Hz to 16 kHz. The surgical mask (*M*2) and the N95 mask (*M*3) showed similar performances with overall attenuations equal to 2.3 dB and 2.9 dB, respectively.

### E. Statistical analyses

Generalized linear mixed models (GLMMs) were used for the statistical analyses, using the software *R*3.6.0 and the lme4 package.^18^ Different models have been built for the three response variables. GLMMs with a binomial distribution (Laplace approximation) are usually used to model binary outcome variables. The log odds of the outcomes are modeled as a linear combination of the predictor variables when data are clustered or there are both fixed and random effects. In this study, the binary outcomes were SI and LE; the SI response outcome in the model was coded with a binary score (0/1 corresponding to a wrong/correct response), whereas the LE response variable was divided by 100 to restrain the range between 0 and 1. The independent variables used were mask (4 levels) and room (2 levels). Random factors are best defined as noise in the data. These are effects that arise from uncontrollable variability within the sample. Subject level variability often is a random effect. In the proposed models, the listener and type of headphones were used as random factors.

Tukey's *post hoc* pair-wise comparisons were performed to examine the differences between all levels of the mask factor. These are pair-wise *z* tests, where the *z* statistic represents the difference between an observed statistic and its hypothesized population parameter in units of the standard deviation. The *p*-values for these tests were adjusted using the default single-step method.^19^ The GLMM outputs include the estimates of the fixed effects coefficients, the standard error associated with the estimate, the test statistic, *z*, and the *p*-value. From the estimates, it is possible to calculate the odds ratio (OR) as the exponential function of the estimate. An OR is a measure of association between an exposure and an outcome. The OR represents the odds that an outcome will occur given a particular exposure compared to the odds of the outcome occurring in the absence of that exposure.

## III. RESULTS

To evaluate the effect of the different amplitude responses of the headphones on the SNR, we first searched online to obtain the amplitude response of all the identifiable headphones reported by the listeners. Next, the following calculations were performed: (1) the relative spectra of the speech material concatenated without pauses in the four masks conditions, (2) the relative spectrum of the noise used as masker to obtain a SNR of 3 dB, (3) the amplitude response of all identifiable headphones reported by the listeners was applied to the noise and the speech spectra, and (4) the SNR was recalculated. We were able to find the amplitude responses of headphones used by 28 of the 40 participants. The average SNR among all the evaluations performed was 2.986 dB with a standard deviation of 0.052 dB.

**FIG. 3.**
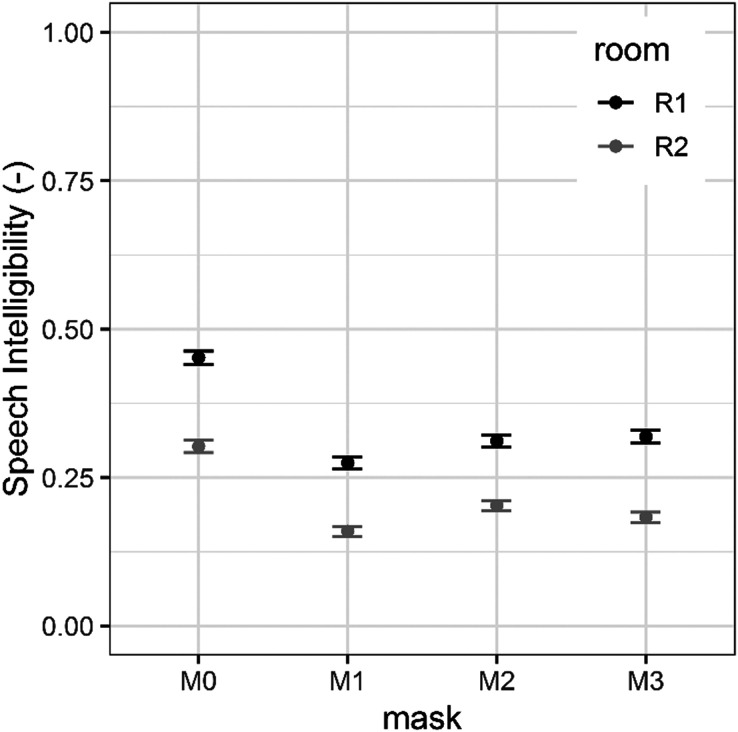
Mean SI across listeners in the conditions with and without masks in the two rooms. Error bands indicate ± standard error.

**FIG. 4.**
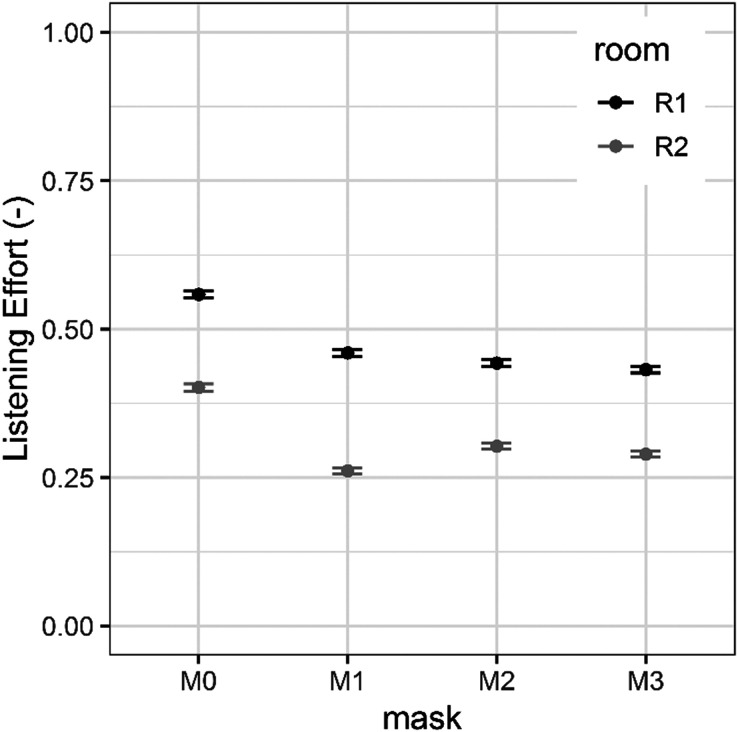
Mean LE rated from extremely difficult to extremely easy across listeners in the conditions with and without masks in the two rooms. Error bands indicate ± standard error.

A GLMM fit by maximum likelihood with a binomial distribution (Laplace approximation) was used for the SI and LE results. The following two predictors were considered: mask and room. The mask predictor consisted of four factors (*M*0 = no mask, *M*1 = fabric mask, *M*2 = surgical mask, *M*3 = N95 mask). The second predictor, room, was a factor with two levels: room 1 and room 2. Room 1 represents the room with a short reverberation time and room 2 represents the room with a long reverberation time. The random factors included in the model were listener and headphones. Model results of the SI and LE are shown in Table I, whereas their mean values and standard errors are reported in Fig. 3 for SI and in Fig. 4 for LE.

**TABLE I.** Generalized linear mixed effect model (binomial family) for response variables SI and LE (from extremely difficult to extremely easy) considering as predictors (1) mask and (2) room. Listener and type of headphones were used as random factors. Significance codes for the *p*-values: *** < 0.001, ** < 0.01, * < 0.05.

SI (–)	Estimate	Standard error	*z* value	*p*-value	
(Intercept)	−0.24	0.17	−1.41	0.159	
Mask *M*1	−0.84	0.05	−16.16	<0.001	***
Mask *M*2	−0.61	0.05	−11.99	<0.001	***
Mask *M*3	−0.64	0.05	−12.61	<0.001	***
Room 2	−0.70	0.04	−18.56	<0.001	***

LE (–)	Estimate	Standard error	*z* value	*p-*value	
(Intercept)	0.44	0.15	3.07	0.002	**
Mask *M*1	−0.95	0.06	−16.94	<0.001	***
Mask *M*2	−0.89	0.06	−16.06	<0.001	***
Mask *M*3	−1.00	0.06	−17.82	<0.001	***
Room 2	−1.37	0.04	−32.78	<0.001	***

Regarding the SI model, the estimate of standard deviations for random effects was 0.59 for listener and 0.09 for headphones. This means that the variability introduced by the use of different types of headphones was smaller than the inter-listener variability. The probability of correctly recognizing a word in room 1 was 50% higher than the probability of correctly recognizing a word in room 2 (*Χ*^2^ = −0.70, OR = 0.50, *p <* 0.001). Participants were able to correctly recognize 34% of the words in the room 1 condition and 21% of the words in the room 2 condition, averaging across all the mask conditions. When the speech was presented in the presence of *M*1 (the fabric mask), the probability of correctly recognizing the words was 57% less than in the unmasked condition (*Χ*^2^ = −0.85, OR = 0.43, *p <* 0.001). When the speech was presented in the presence of *M*2 (the surgical mask), the probability of correctly recognizing the words was 46% less than in the unmasked condition (*Χ*^2^ = −0.61, OR = 0.54, *p <* 0.001). Finally, when speech was presented in the presence of *M*3 (the N95 mask), the probability of correctly recognizing the words was 47% less than in the unmasked condition (*Χ*^2^ = −0.64, OR = 0.53, *p <* 0.001).

As shown in Table II, *post hoc* comparisons confirmed that, overall, the differences in SI among the four types of masks were statistically significant except for the difference between the surgical (M2) and N95 (M3) masks. Regarding the LE model, the estimate of standard deviations for random effects was 0.88 for listener and 0.01 for headphones. The variability introduced by the use of different headphones was again smaller than the inter-listener variability. For the participants, the probability of experiencing greater LE in room 2 compared to room 1 was 75% (*Χ*^2^ = −1.37, OR = 0.25, *p <* 0.001). Compared to the speech recorded without a mask, the probability of experiencing greater LE was 61% when the speech was recorded with the fabric mask (*Χ*^2^ = −0.95, OR = 0.39, *p <* 0.001), 59% for the surgical mask (*Χ*^2^ = −0.89, OR = 0.41, *p <* 0.001), and 63% for the N95 mask (*Χ*^2^ = −1.00, OR = 0.37, *p <* 0.001). As shown in Table II, *post hoc* comparisons confirmed that, overall, the difference in LE was statistically significant only when comparing the condition without a mask to the conditions with masks. However, the LE did not differ significantly as a function of mask type.

**TABLE II.** Multiple comparisons of means using Tukey contrasts for the predictive variables SI and LE for the factor mask. Significance codes for the *p*-values: *** < 0.001, ** < 0.01, * < 0.05.

SI (–)	Estimate	Standard error	*z* value	*p*-value	
*M*1–*M*0	−0.848	0.052	−16.165	<0.001	***
*M*2–*M*0	−0.610	0.051	−11.999	<0.001	***
*M*3–*M*0	−0.644	0.051	−12.619	<0.001	***
*M*2–*M*1	0.238	0.055	4.359	<0.001	***
*M*3–*M*1	0.204	0.055	3.721	0.001	**
*M*3–*M*2	−0.034	0.053	−0.640	0.918	

LE (–)	Estimate	Standard error	*z* value	*p*-value	
*M*1–*M*0	−0.953	0.056	−16.964	<0.001	***
*M*2–*M*0	−0.895	0.056	−16.060	<0.001	***
*M*3–*M*0	−1.004	0.056	−17.818	<0.001	***
*M*2–*M*1	0.058	0.058	1.002	0.748	
*M*3–*M*1	−0.050	0.059	−0.862	0.824	
*M*3–*M*2	−0.109	0.058	−1.869	0.242	

## IV. DISCUSSION

Evaluation of the sound attenuation per octave band for each mask revealed that the greatest attenuation was obtained for frequencies higher than 2000 Hz; these frequencies are the most important frequencies for speech understanding. With an overall attenuation of 4.2 dB, the fabric mask (*M*1) was the most attenuating. The N95 mask (*M*3) and surgical masks (*M*2) produced similar levels of attenuation at 2.9 dB and 2.3 dB, respectively.

The sound attenuation results are in line with the scores from the SI test results. Each of the masks was found to have a negative impact on SI, and SI was the highest in the unmasked condition. When compared to the unmasked condition, the speech was 12% less intelligible with the surgical mask, 13% less intelligible with the N95 mask, and 16% less intelligible with the fabric mask. The differences between the four types of masks were statistically significant except for the comparison between the surgical and N95 masks. Performance in the latter two conditions was similar. This is consistent with the results of Palmiero *et al.*^9^ in which high scores of SI were obtained with both the N95 and surgical masks. Palmiero *et al.*^9^ also found that the fabric mask was the most degrading for intelligibility.

The SI results are consistent with those of Wittum *et al.*^6^ However, they differ from Mendel *et al.*,^4^ who did not find a degrading effect of the masks on speech understanding, probably because the listening conditions used were too good. In fact, their scores showed a ceiling effect with intelligibility higher than 96.9% in all of the conditions.

In terms of the LE, ratings were similar across mask types. When compared to the reported LE without a mask, speech produced with the surgical mask was 11% more difficult to listen to, followed by the fabric mask (12%), and finally the N95 mask (13%). However, the LE was not statistically different when comparing the three conditions with masks.

Reverberation time also was a strongly significant factor for the SI scores. In the quiet condition, average SI scores were 34% for the room with the short reverberation time (room 1, 0.4 s) and 21% for the room with the longer reverberation (room 2, 3.1 s). LE also was influenced by reverberation time. Participants rated the LE as 16% less difficult for stimuli in the room with shorter reverberation times (room 1) compared to ratings for the room with longer reverberation times (room 2).

The listeners involved in this study were young adults with normal hearing. Future research will also involve students with hearing problems. A limitation of the methodological approach was the fact that the listeners used different headphones. The use of different headphones could affect the SNR because of the spectral differences between speech and noise. However, the use of the mixed-effects model allowed control of the aforementioned variability. The variability introduced in the SNR with the headphones reported by the participants was 0.052 dB. To avoid this confounding factor in future studies, the speech-shaped noise should have the same spectral shape of the speech material used. In this way, the amplitude response of the headphones will equally affect the noise and the speech. Finally, this test was conducted using CVC words without taking into account the effect of context on a whole sentence. In fair or poor acoustics conditions, sentence intelligibility can reach a performance that is 30% higher than performance obtained using CVC words.^20^ Compared to single words, sentences provide linguistic context (i.e., syntactic structure and semantic cues), which may allow listeners to accurately infer words that would otherwise be unintelligible.^21^ For this reason, the next step should be to evaluate the impact of wearing a mask on SI at a sentence level.

## V. CONCLUSIONS

This study aimed to explore the influence of the use of face masks on classroom communication by evaluating which of three types of masks (fabric, surgical, and N95 masks) yielded the highest SI for college students. For this reason, parameters, such as reverberation times (0.4 s and 3.1 s) and speech-shaped noise SNR +3 dB, were used to simulate a real classroom environment, overcoming previous studies focused on the medical field only (i.e., anechoic and/or no noise condition). Because of the problems that Covid-19 is forcing us to face, the significance of this study consists in giving recommendations on the best type of masks to wear while teaching to minimize their negative effect on SI. The results of this study showed that the use of surgical masks or N95 masks, rather than fabric masks, is strongly recommended in teaching environments. The use of surgical and N95 masks can minimize negative effects on SI and the students' LE while protecting instructors and students alike.

## References

[1] H. J. Steeneken and T. Houtgast , “ Mutual dependence of the octave-band weights in predicting speech intelligibility,” Speech Commun. 28(2), 109–123 (1999). 10.1016/S0167-6393(99)00007-2

[2] A. Goldin , B. E. Weinstein , and N. Shiman , “ How do medical masks degrade speech perception?,” Hear. Rev. 27(5), 8–9 (2020).

[3] A. H. Bannwart Dell'Aringa , E. Satico Adachi , and A. R. Dell'Aringa , “ Lip reading role in the hearing aid fitting process,” Rev. Bras. Otorrinolaringol. 73(1), 101–105 (2007). 10.1590/S0034-72992007000100016 PMC9443519 17505606

[4] L. L. Mendel , J. A. Gardino , and S. R. Atcherson , “ Speech understanding using surgical masks: A problem in health care?,” J. Am. Acad. Audiol. 19(9), 686–695 (2008). 10.3766/jaaa.19.9.4 19418708

[5] R. M. Cox , G. C. Alexander , and C. Gilmore , “ Development of the Connected Speech Test (CST),” Ear Hear. 8(5), 119S–126S (1987). 10.1097/00003446-198710001-00010 3678650

[6] K. J. Wittum , L. Feth , and E. Hoglund , “ The effects of surgical masks on speech perception in noise,” in *Proceedings of the International Congresses on Acoustics 2013*, Montreal (2013).

[7] D. N. Kalikow , K. N. Stevens , and L. L. Ellitt , “ Speech perception in noise test (SPIN),” J. Acoust. Soc. Am. 61, 1337–1351 (1977). 10.1121/1.381436 881487

[8] R. Saeidi , T. Niemi , H. Karppelin , J. Pohjalainen , T. Kinnunen , and P. Alku , “ Speaker recognition for speech under face cover,” in *Proceedings of Interspeech 2015*, Dresden (2015).

[9] A. J. Palmiero , D. Symons , J. W. Morgan III , and R. E. Shaffer , “ Speech intelligibility assessment of protective facemasks and air-purifying respirators,” J. Occup. Environ. Hyg. 13(21), 960–968 (2016). 10.1080/15459624.2016.1200723 27362358 PMC5065390

[10] J. S. Bradley , R. D. Reich , and S. G. Norcross , “ On the combined effects of signal-to-noise ratio and room acoustics on speech intelligibility,” J. Acoust. Soc. Am. 106(4), 1820–1828 (1999). 10.1121/1.427932 10530010

[11] A. Astolfi , P. Bottalico , and G. Barbato , “ Subjective and objective speech intelligibility investigations in primary school classrooms,” J. Acoust. Soc. Am. 131(1), 247–257 (2012). 10.1121/1.3662060 22280588

[12] M. L. G. Lecumberri , M. Cooke , and A. Cutler , “ Non-native speech perception in adverse conditions: A review,” Speech Commun. 52(11-12), 864–886 (2010). 10.1016/j.specom.2010.08.014

[13] I. Lehiste and G. E. Peterson , “ Linguistic considerations in the study of speech intelligibility,” J. Acoust. Soc. Am. 31(3), 280–286 (1959). 10.1121/1.1907713

[14] G. E. Peterson and I. Lehiste , “ Revised CNC lists for auditory tests,” J. Speech Hear. Disord. 27(1), 62–70 (1962). 10.1044/jshd.2701.62 14485785

[15] E. Thorndike and I. Lorge , “ The teacher's word book of 30,000 words,” ( Columbia University Teachers College, New York, 1944).

[16] Auditory Potential LLC , “ Minimum Speech Test Battery (MSTB) for adult cochlear implant users” (Auditory Potential LLC, Goodyear, AZ, 2011).

[17] T. Houtgast , H. J. M. A. Steeneken , and R. Plomp , “ Predicting speech intelligibility in rooms from the modulation transfer function – I. General room acoustics,” Acustica 16, 60–72 (1980).

[18] D. Bates , M. Maechler , B. Bolker , S. Walker , and R. Haubo Bojesen Christensen , “ lme4: Linear mixed-effects models using Eigen and S4. R package,” J. Stat. Softw. 67, 1–48 (2014).

[19] T. Hothorn , F. Bretz , P. Westfall , R. M. Heiberger , A. Schuetzenmeister , S. Scheibe , and M. T. Hothorn , “ *R* package ‘multcomp’. Simultaneous inference in general parametric models” (Project for Statistical Computing, Vienna, Austria, 2016).

[20] IEC 60268-16-2003: *Sound System Equipment—Part 16: Objective Rating of Speech Intelligibility by Speech Transmission Index* ( International Electrotechnical Commission, Geneva, Switzerland, 2003).

[21] K. M. Allison , Y. Yunusova , and J. R. Green , “ Shorter sentence length maximizes intelligibility and speech motor performance in persons with dysarthria due to amyotrophic lateral sclerosis,” Am. J. Speech-Lang. Pat. 28(1), 96–107 (2019). 10.1044/2018_AJSLP-18-0049 PMC6503867 31072158

